# Giant bladder stone with squamous cell carcinoma of bladder: Case report and the literature review

**DOI:** 10.1016/j.ijscr.2021.01.082

**Published:** 2021-01-22

**Authors:** Septa Surya Wahyudi, Achmad Romy Syahrial Rozidi, Rahmat Sayyid Zharfan, Dewi Setyowati

**Affiliations:** a Faculty of Medicine University of Jember, Jember, 68121, Indonesia; b Faculty of Medicine Universitas Airlangga, Dr. Soetomo General Hospital, Surabaya, 60286, Indonesia; c Midwifery Department, Faculty of Medicine Universitas Airlangga, Dr. Soetomo General Hospital, Surabaya, 60286, Indonesia

**Keywords:** Bladder cancer, Case report, Giant bladder stone, Squamous cell carcinoma

## Abstract

**Introduction and importance:**

Concurrent bladder neoplasm and giant bladder stone are rare in contemporary urological practice. Squamous cell carcinoma (SCC) is rare histologic diagnosis of bladder cancer.

**Case presentation:**

A 45 y.o. male, with lower abdominal pain when urinating, that comes and goes in the last 35 years. He had gross hematuria a year ago. The patient comes from a rural region, which undiagnosed for years. Physical examination showed a suprapubic abdominal solid mass, sized 20 × 10 cm, without tenderness. On plain radiography, showed radiopaque lesion which fully occupies the bladder. The ultrasound showed bilateral hydronephrosis. The patient underwent vesicolithotomy, and a giant bladder stone (size of 14 × 9 cm) was found, with incidental finding of suspicious malignant mass. The patient refuses radical cystectomy. Due to mass characteristics that are manageable for complete excision and the need for histopathological studies, bladder preservative therapy was applied with complete tumor excision and biopsy. The mass pathological diagnosis is grade 2 squamous cell carcinoma with lamina muscularis invasion, staged pT3bN0M0. The patient underwent cisplatin-based chemotherapy, with regular evaluation. The possibility of future radical cystectomy remains open.

**Clinical discussion:**

By diameter, the stones found in our patient is perhaps one of the largest that ever reported being associated with bladder SCC. The bladder stones causing chronic mucosal injury, lead to the development of SCC. In limited situation, bladder preservation therapy may be considered for muscle-invasive bladder cancer.

**Conclusion:**

Despite its rarity, SCC along with the chronic bladder stone is possible, and needs more attention.

## Highlights

• Concurrent giant bladder stones and bladder squamous cell carcinoma (SCC) is rare.
• Recurrent urinary tract infections should be suspected as bladder stones.
• The giant size of bladder stone makes open surgery the only therapeutic modality.
• Chronic inflammation due to bladder stones may contribute to the development of SCC.
• In a limited situation, bladder preservative therapy may be considered for MIBC.

## 1 Introduction

Urinary tract stones (urolithiasis) are a common disease in the world. Urolithiasis can cause symptoms of pain, bleeding, obstruction of urinary flow, or infection. The epidemiological study states the prevalence of urolithiasis is between 4 and 20% in developing countries [1]. These stones can form in the kidneys, ureter, or bladder. The bladder stones itself responsible for 5% of urinary tract stones [2].

Squamous cell carcinoma (SCC) is the rare histopathological diagnosis of the bladder which is only 2%–3% from all bladder cancers. Concomitant bladder stones with bladder tumors are even rarer [3]. We aim to report this rare clinical presentation may develop, then it should become a concern for urologists. We report a case of giant bladder stones that are incidentally associated with bladder cancer.

This work has been reported in line with the SCARE criteria [4].

## 2 Presentation of case

A 45 years old male, referred to our urology outpatient clinic (secondary referral hospital), with the complaint of lower abdominal pain when urinating (dysuria), for the last 35 years. He had gross hematuria a year ago. There was no history of trauma, no change in appetite, no weight loss, no fever, or other systemic symptoms. The patient is a farmer. He lives in a rural region. He had visited the local public health center for several years, and diagnosed with recurrent urinary tract infection, then received medication such as analgesics, and antibiotics. He is a smoker but never consume alcohol. He had not been worked in a chemical environment. The patient has no previous history of urolithiasis, malignancy, allergy, or other systemic disease. There is no history of cancer in the family.

**Fig. 1:**
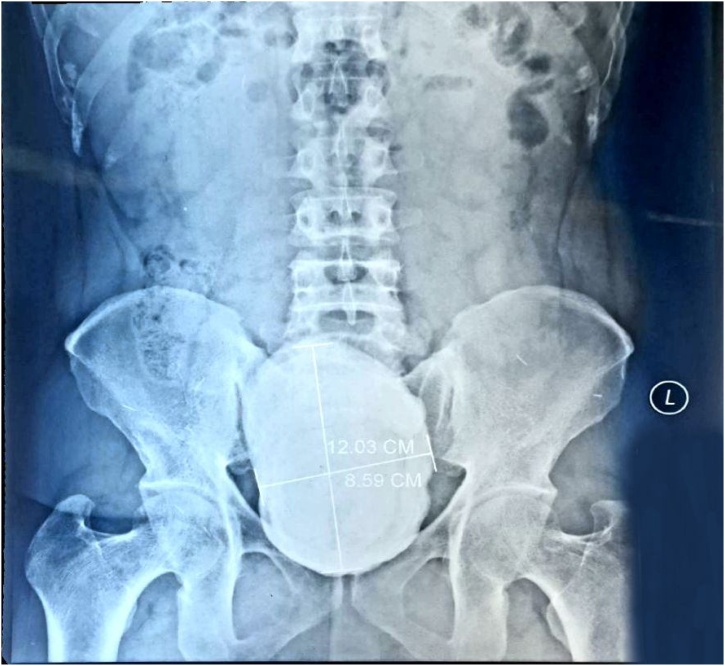
The patient’s BOF radiography shows a radiopaque round shape that occupies the bladder.

**Fig. 2:**
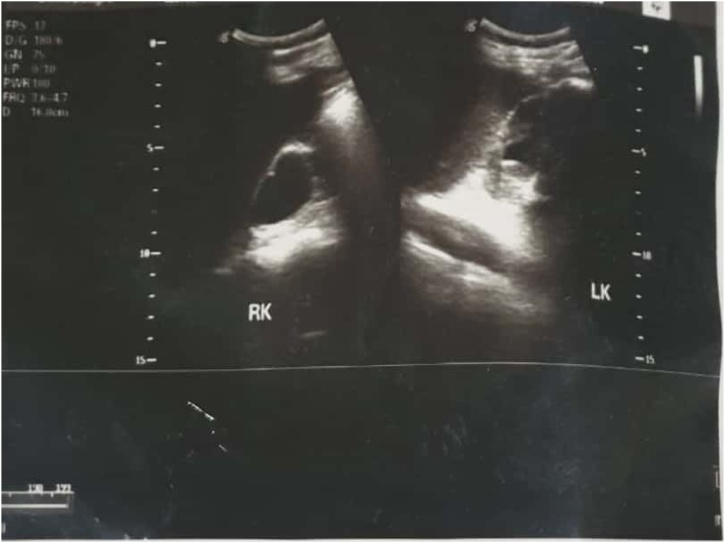
Ultrasound examination revealed bilateral hydronephrosis (severe hydronephrosis of the right kidney; and moderate hydronephrosis of the left kidney). RK: Right Kidney; LK: Left Kidney.

On physical examination, it showed an abdominal mass, in the suprapubic region with size of 20cm × 10cm. The mass is solid, round, distinct border, mobile, and without tenderness in palpation. No prostate enlargement was found on digital rectal examination. Urine examination showed signs of hematuria. There is radioopaque lesion in the bladder region on the plain abdominal radiograph. The lesion is round, single, large, occupies the entire bladder (Fig. 1). It was most likely to be a giant bladder stone. The ultrasound examination of the kidney revealed severe right hydronephrosis, and moderate left hydronephrosis (Fig. 2). Laboratory assessment shows impaired kidney function test.

**Fig. 3:**
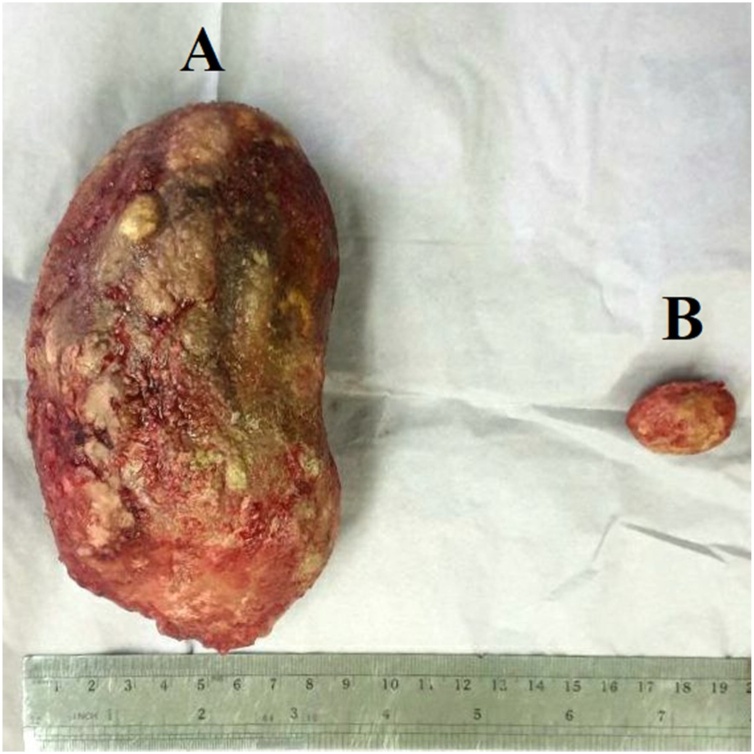
The giant bladder stone with a size of 14 cm × 9 cm (A). Furthermore, we found a mass of the suspicious tumor, sized 3.5cm × 2.5cm × 1.5 cm (B).

**Fig. 4:**
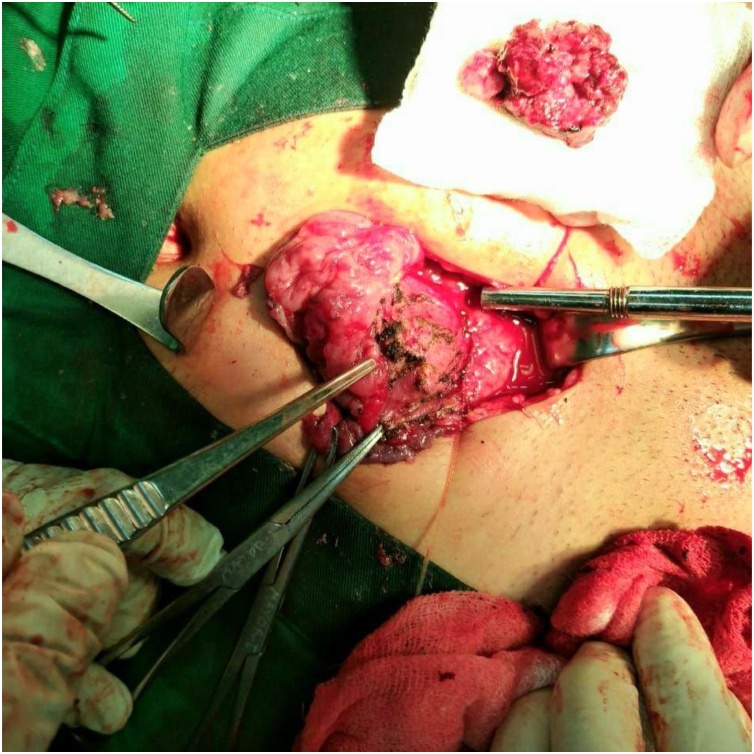
Tumor exicision and biopsy procedure.

The patient underwent vesicolithotomy under regional anesthesia. The procedure was performed by urologist with experience of more than 5 years. During the operation, a giant bladder stone was found, with size of 14cm × 9cm (Fig. 3A). Furthermore, we incidentally found suspicious malignant mass over the bladder's postero-lateral wall. The mass was nodular, white, irregular surface, crumbly, fixed to surrounding tissue, with size of 3.5cm × 2.5 cm × 1.5 cm (Fig. 3B). During the vesicolithotomy procedure, we planned radical cystectomy, but the patient refused. Eventually, we did bladder preservative therapy with complete tumor excision and biopsy sampling (Fig. 4). Several factors that are taken into our consideration are solitary tumors, small-sized, favourable bladder function, no lymph nodes metastases, manageable for complete excision, and the need for histopathological studies. Due to limited facilities, we got the histopathological result on the next day. Its revealed grade 2 squamous cell carcinoma with invasion into the lamina muscularis. The pathological diagnosis was squamous cell carcinoma invading the muscle tissue. The tumor was staged as pT3bN0M0 as the AJCC 7th edition staging system.

The patient was observed in 3 days post-surgical procedure, to ensure there is no leakage. The post-operative complaint of the patient is only pain on the surgical site. On follow up visit, the patient had no significant clinical complaints, along with corrected kidney function test. We explore the patient's perspective on the plan of radical cystectomy, urinary diversion, postoperative recovery, and highlight possible risks during the procedure. The patient and family are involved in the decision making after receiving adequate information, but he still refuses the radical cystectomy. Then, it was decided to reconsider the treatment with adjuvant chemotherapy.

The strict follow-up was carried out while considering and discussing the possibility of a radical cystectomy in the future. We periodically evaluate the clinical feature (the sign of hematuria, mass, and metastasis), radiological assessment (including chest CT Scan), periodic surveillance, and cystoscopy. The underlying factors that influence patient’s adherence are anxiety about recurrence, cost, and the inconvenience of certain procedure. Currently, the patient underwent a gemcitabine-cisplatin regimen of chemotherapy. The patient voided normally with normal urine output, despite the mild urinary tract symptoms, which improves within several weeks. The patient remained asymptomatic, with no evidence of any residual or recurrent disease, for the last three months.

## 3 Discussion

Bladder stones are more common in adults; the male is more affected [1]. Recurrent urinary tract infections, urinary retention, and hematuria are common signs of this disease [2]. Our patient develop giant bladder stone, due to long period of delayed diagnosis. Diagnostic procedures used to confirm the presence of bladder stones are ultrasound, plain abdominal radiograph, or CT scan.

Bladder stone treatment are based on the stone size, location, density, and the number of stones. In large stones, open surgical removal of stones is the first choice [5]. There is currently no size classification used for bladder stones; its called large if the patient is unable to pass them through the urethra. In patients with bladder stones >2 cm in size, it was recommended to remove stones through an open surgical procedure, especially on hard stones. However, bladder stone with sizes above 2 cm, it will be difficult to break it down. Recurrence after stone removal surgery is rare [6].

Squamous cell carcinoma (SCC) accounts for only <5% of bladder cancers. Male, and smoking remains the major risk factor. Bladder cancer is associated to chronic inflammation process which contributes to squamous cell metaplasia, and dysplasia [7]. The bladder stones resulting in chronic mucosal injury that induce the development of SCC [8]. In our case, the SCC is invading the lamina muscularis (muscle tissues). The main treatment of muscle-invasive bladder cancer (MIBC) is radical cystectomy [9]. In limited situation, urologist may face several conditions such as: patient is unfit for surgery, the consideration to avoid cystectomy morbidity, or patient refusal. Bladder preservation therapy can be an alternative treatment option while maintaining the patient's quality of life. The ideal criteria for bladder preservation therapy including: solitary mass, low volume, stage of T2 or below, absence of carcinoma in situ, no hydronephrosis, and maximal resection with routine surveillance [10]. Additional therapeutic modalities such as chemotherapy and radiation therapy regimens may improve bladder preservation therapy outcomes [10]. Our case did not fully meet those ideal criteria, due to T3b in staging, with bilateral hydronephrosis. So, we still considering the radical cystectomy procedure, while conducting adjuvant chemotherapy (gemcitabine/cisplatin), and strict monitoring. The comprehensive review from Hamad et al. [10], states that patient refusal is one of the main factors in the decision of radical cystectomy, refers to the Southwest Oncology Group phase II trial which states that 45% of MIBC patients do not undergo radical cystectomy because of patient refusal, in addition to other causes such as: unresectable tumors (34%), and surgically or medically unfit (21%).

Neoadjuvant chemotherapy leads to stage reduction of the bladder tumor. Radiotherapy as single modality has poor local control in bladder SCC [11]. In current literature, management strategies of combining radiotherapy and chemotherapy shows various outcomes. SCC is considered a chemotherapy-resistant disease, but there is study that show high response rate with moderate toxicity profile of neoadjuvant chemotherapy (gemcitabine/cisplatin) in bladder SCC. Radiotherapy with concurrent chemotherapy also useful alternative for unresectable bladder cancer, or in bladder preservation therapy [12]. The EAU guideline also states varied outcome (partial and complete) local response to cisplatin-based chemotherapy is the alternative therapy for highly selected patients in bladder-sparing treatment MIBC. However, radiotherapy is not recommended for stages above T2 [13].

Based on available literature [3,8,[14], [15], [16], [17], [18], [19], [20]], three case reports state a mass in the urinary bladder with bladder stone has the biopsy with the squamous cell carcinoma [3,8,16], while others reported translational cell carcinoma, urothelial carcinoma, hemangioma, and villous adenoma. By diameter, the stones found in our patient's bladder are perhaps the largest which ever reported being associated with bladder SCC. Kirakoya et al., reported similar case to ours, a patient with bladder stones of 13 cm × 10 cm with muscle-invasive SCC. This patient also refused RC, then died 18 months later due to loss to follow-up, and had not received chemotherapy or radiotherapy [3]. Of the three literature of bladder stone with bladder SCC, only Aboutaleb et al. [16] who successfully performed radical cystectomy with ileal conduit urinary diversion, but there is no reported follow-up (Table 1).

**Table 1:** Summary of case reports of bladder mass diagnosis and management.

Author, year	Age	Sex	Clinical presentation	Method of diagnosis/treatment	Bladder Stone	Biopsy	Pathology	Chemotherapy	Radiotherapy	Follow up
Sheehan, et al. [14] 2015	18	F	Haematuria; nonspecific intermittent abdominal pains	Urinary tract ultrasound: echo dense polypous lesion present on the bladder, sized 21 × 15 × 17 mm;	NA	Yes	Grade 2 papillary transitional cell carcinoma of the bladder, no lamina propria invasion (G2pTa TCCB)	Intravesical mitomycin C	NA	Shown no signs of recurrence on cystoscopy, at 3rd and 6th month
				Rigid cystoscopy and transurethral resection of the unifocal 2 cm × 2 cm bladder tumor						
Nabbout et al. [15] 2013	28	M	Gross haematuria; and left flank pain	Computerized tomography of abdomen-pelvis without contrast due to elevated creatinine: mass with diameter of 6 cm on bladder posterior wall extending to the left ureteral orifice; left hydronephrosis; without lymphadenopathy.	NA	Yes	T2a N0, urothelial carcinoma	NA	NA	No recurrence until the 6th month of follow up
				Cystoscopy: Large masses, multiple, on the left part of the trigone extend to the prostatic urethra;						
				A left nephrostomy tube placement followed by TURBT						
Fernando et al. [8] 2017	57	M	dysuria, intermittent flow, nocturia, haematuria	KUB plain radiograph showed lesion suspected bladder stone;	A large bladder stone with a size of 5.5 cm × 5.6 cm	Yes	Well-differentiated squamous cell carcinoma (SCC) invading lamina propria and lamina muscularis propria.	NA	NA	Patient died 9 months after diagnosis
				USG: echogenic lesion, large-sized, irregular mass, internal vascularity on the left lateral wall of bladder			Absent of lymph vascular invasion.			
				Patient underwent vesicolitholapaxy + TURBT						
				Offered radical cystectomy - abandoned						
Aboutaleb et al. [16] 2011	55	F	Severe dysuria	Urinalysis: pyuria, microscopic haematuria, mild proteinuria; on urine culture show the presence of *Escherichia coli*;	Stone with size 5 cm × 6 cm	Yes	Muscle invasive SCC	No	No	NA
				Abdomen - pelvic USG + non-contrast CT: contracted bladder with diffuse wall thickening, bladder stone with size of 5 cm × 6 cm, bilateral hydronephrosis, hepatomegaly.						
				KUB: large, lamellated radiopaque shadow at the pelvis;						
				VCUG revealed left reflux grade IV;						
				Cystoscopy: stone with underlying multiple erythematous bladder wall;						
				Radical cystectomy + ileal conduit urinary diversion was conducted						
Kirakoya et al. [3] 2018	60	M	Dysuria; haematuria; nocturia, intermittent suprapubic pain; a hard well-defined suprapubic mass; digital rectal examination: a hard and painless mass	Pyuria; plain abdominal x-ray: opacity occupying the entire pelvic.	Whitish weighed 1.1 kg with 13 cm × 10 cm	Yes	Muscle invasive squamous cell carcinoma (SCC)	NA	NA	lost to follow up for 18 months; recurrent haematuria; died from severe anaemi
				Cystolithotomy						
				The patient refuses radical cystectomy						
Prelaj et al. [17] 2016	71	M	Dysuria; haematuria,	Cystectomy + lymphadenectomy + placement of bilateral ureterocutaneostomy;	No	Yes	pT2 high-grade urothelial carcinoma	Yes	NA	Underwent neoadjuvant chemotherapy
				On the left lateral, the anterior wall was found bladder vegetative lesion with size of 34 × 24 mm						
Syu, Syuan-hao et al. [18] 2018	17	F	persistent gross hematuria	Partial resection of TURBT showed chronic inflammation; 3.6 cm mass on supero-anterior wall of bladder	No	Yes	Cavernous hemangioma	NA	NA	Showed no local recurrence in 2 years (6, 12, and 24 months follow up) on Cystoscopy and CT
Morozumi et al. [19] 2017	62	M	Macrohematuria; and dysuria	Cystoscopy: multiple nodular tumors,	No	Yes	Sarcomatoid variant of invasive Urothelilal Carcinoma, pT4aN0M0, pStage IV, anti-G-CSF immunostaining (positive)	Yes	NA	One month post surgery: appeared subcutaneous and multiple liver metastases ap
				Transurethral biopsy: Urothelial Carcinoma.						On 9th months: the patient died
Kato, Yoichiro et al. [20] 2013	85	F	painless gross hematuria	Cystoscopy: two papillary tumors in the bladder;	No	Yes	Villous adenoma, and urothelial carcinoma	NA	NA	Follow up on 3rd month: the cystoscopy found a small papillary tumor in the trigone.
				Ultrasonography: mass on the right bladder wall with a diameter of 15 mm;						
				CT scan: 16 mm mass on the right wall, and 9 mm mass on the left wall, with enhancement.						At 24th months follow-up: no local recurrences were detected.

## 4 Conclusion

A giant bladder stone is a rare disease in modern urology clinical practice. In some conditions, the bladder stone symptoms are nonspecific and often asymptomatic, which leads to an undiagnosed case. The very large stone size makes open surgery the only alternative therapy. The bladder stones may cause chronic mucosal injury leading to the development of SCC. Although concurrent findings of bladder stones and SCC bladder are very rare, this condition is still possible. This topic requires further discussion and evaluation, particularly in its management.

## Declaration of Competing Interest

The authors report no declarations of interest.

## Sources of funding

None declared.

## Ethical approval

Ethical approval was obtained from the Research Ethics Committee of the concerned hospital.

## Consent

Written informed consent was obtained from the patient for publication of this case report and accompanying images. A copy of the written consent is available for review by the Editor-in-Chief of this journal on request.

## Author contribution

Septa Surya Wahyudi: the person who involved in patient care, study supervision. Achmad Romy Syahrial Rozidi: study design, data collection, and writing manuscript. Rahmat Sayyid Zharfan: writing manuscript, and layout editing. Dewi Setyowati: writing manuscript, and review of the literature.

## Registration of research studies

Not applicable.

## Guarantor

Septa Surya Wahyudi, MD.

## Provenance and peer review

Not commissioned, externally peer-reviewed.
